# *Pseudomonas aeruginosa* Is More Tolerant Under Biofilm Than Under Planktonic Growth Conditions: A Multi-Isolate Survey

**DOI:** 10.3389/fcimb.2022.851784

**Published:** 2022-02-28

**Authors:** Janne G. Thöming, Susanne Häussler

**Affiliations:** ^1^ Department of Clinical Microbiology, University Hospital Copenhagen, Rigshospitalet, Copenhagen, Denmark; ^2^ Molecular Bacteriology, Twincore Center for Experimental and Clinical Infection Research GmbH, Hannover, Germany; ^3^ Molecular Bacteriology, Helmholtz Centre for Infection Research, Braunschweig, Germany; ^4^ Cluster of Excellence RESIST (EXC 2155), Hannover Medical School, Hannover, Germany

**Keywords:** conditional tolerance, biofilms, *Pseudomonas aeruginosa*, biofilm-associated chronic infections, clinical isolates, antimicrobial susceptibility testing (AST), biofilm-induced tolerance, antibiotic treatment

## Abstract

Biofilm-associated bacteria exhibit profound changes in bacterial physiology. They thrive in the environment but also in the human host in protected sessile communities. Antimicrobial therapy usually fails, despite the absence of genotypic resistance, and it is commonly accepted that biofilm-grown bacteria are up to 1,000-fold more resistant than planktonic cells. We are only at the beginning to understand the reasons for biofilm recalcitrance, and systematic approaches to describe biofilm-induced tolerance phenotypes are lacking. In this study, we investigated a large and highly diverse collection of 352 clinical *Pseudomonas aeruginosa* isolates for their antimicrobial susceptibility profiles under biofilm growth conditions towards the antibiotics ciprofloxacin, tobramycin, and colistin. We discovered characteristic patterns of drug-specific killing activity and detected conditional tolerance levels far lower (in the range of the minimal inhibitory concentration (MIC)), but also far higher (up to 16,000-fold increase compared to planktonic cells) than generally believed. This extremely broad distribution of biofilm-induced tolerance phenotypes across the clinical isolates was greatly influenced by the choice of the antibiotic. We furthermore describe cross-tolerance against ciprofloxacin and tobramycin, but not colistin, and observed an additive activity between biofilm-induced tolerance and genetically determined resistance. This became less evident when the biofilm-grown cells were exposed to very high antibiotic concentrations. Although much more remains to be learned on the molecular mechanisms underlying biofilm-induced tolerance, our data on intra-species variations in tolerance profiles provide valuable new insights. Furthermore, our observation that colistin appears to act independently of the tolerance mechanisms of individual clinical strains could make colistin a valuable therapeutic option in chronic biofilm-associated infections characterized by the presence of particularly tolerant strains.

## Introduction

Frequent use of antibiotics favors bacterial strains that have acquired mechanisms to overcome drug inhibition and lethality. Various genetically inherited mechanisms have been identified in bacteria that reduce the efficacy of a drug. These include, for example, mutations of the target structures, enzymatic inactivation of the antibiotic, or the reduction of its intracellular concentrations through reduced influx and/or increased efflux. The phenotypic consequences of genetically conferred resistance are usually monitored by the use of standard antimicrobial susceptibility testing (AST), which largely relies on the determination of the minimal inhibitory concentration (MIC) values of the antibiotic. While the assessment of MICs has a significant impact on the choice of a therapeutic strategy, it has been argued that the predictive power of MIC measurements is less suitable for the implementation of successful treatment regimens in patients with chronic, biofilm-associated infections (Smith et al., 2003). The ability of bacteria to survive a transient exposure of high antibiotic concentrations in a non-specific way, for example when the bacteria temporarily stop growing, is commonly described as tolerance and might be more relevant than inherited drug resistance, when it comes to describing the inability of our currently used drugs to combat biofilm-associated infections.

The biofilm mode of growth enables bacteria to evade the attacks from the immune system and to survive exposure to high concentrations of antimicrobial agents. It is commonly accepted that bacteria exhibit an up to 1,000-fold increased tolerance towards a broad range of different classes of antibiotics under biofilm- as opposed to planktonic growth conditions (conditional tolerance) (e.g. Costerton et al., 1987; Lewis, 2001; Lebeaux et al., 2014; Hall and Mah, 2017; Ciofu and Tolker-Nielsen, 2019). Of note, this does not seem to be true for all antibiotics when the tolerance profile of biofilm-grown cells is compared with that of planktonic cells from the stationary phase (Spoering and Lewis, 2001). However, despite the great attention and clinical importance, the reason for the recalcitrance of biofilm-grown bacteria is only incompletely understood and there is only a limited number of studies on the biofilm-induced tolerance profile of a larger panel of bacterial isolates (Ceri et al., 1999; Moskowitz et al., 2004; Müsken et al., 2017).

In this study, we investigated overall 352 clinical *Pseudomonas aeruginosa* isolates and determined their susceptibility profiles against the three commonly used antibiotics tobramycin, ciprofloxacin, and colistin under biofilm growth conditions. We describe an extremely broad distribution of conditional tolerance phenotypes across the various clinical isolates, which are dependent on the individual strain background and influenced by the choice of the antibiotic. We furthermore describe an additive activity between biofilm-induced tolerance and genetically determined resistance, which however becomes less apparent if the biofilm-grown cells are exposed to very high antibiotic concentrations.

## Methods

### Strains, Media and Growth Conditions

For this study, we selected 352 well characterized clinical *P. aeruginosa* isolates, which have been previously collected across Europe (Hornischer et al., 2019). The strains have been isolated from diverse infection sites, including acute as well as chronic infections (**Electronic Supplementary Table ES1**). Minimal inhibitory concentrations (MIC) of all isolates are publicly available (Khaledi et al., 2020). Biofilm formation was assessed by confocal microscopy in the course of a previous study (Thöming et al., 2020). Bacteria were grown in standard rich medium culture conditions (lysogeny broth, LB) at 37°C. Overnight cultures were incubated with constant shaking (180 rpm); biofilms were grown statically without shaking.

### Antimicrobial Susceptibility Testing Under Biofilm-Growth Conditions

We performed large-scale antimicrobial susceptibility testing of the clinical isolate collection for tobramycin, ciprofloxacin, and colistin under biofilm growth conditions as previously described (Müsken et al., 2010; Donnert et al., 2020). In brief, overnight cultures of clinical isolates were adjusted to an OD_600_ of 0.02 and 100 μl of the bacterial suspension were added to the wells of a sterile half-area, 96-well µClear microtiter plate (Greiner Bio-One). The plate was sealed with an air-permeable BREATHseal cover foil (Greiner Bio-One) and incubated without shaking at 37°C in humid atmosphere. After 24 h, 60 μl of antibiotic solution in diluted LB media were carefully added, resulting in a total volume of 160 µl per well. Antibiotic solutions were adjusted to final concentrations of 1; 4; 16; 64; 256; and 1,024 μg/ml. For the growth control, 60 µl diluted LB media (1:3 in deionized water) without antibiotics were added. The antibiotic exposed biofilms were incubated for 24 h and subsequently resuspended by the use of a multichannel pipette. Tenfold serial dilutions were spotted onto rectangular LB agar plates and incubated at 37°C. Growth was evaluated after 16 h of incubation and further monitored over a period of 48 h to ensure that all surviving bacteria were detected. Colony-forming units (CFU) per ml were calculated for surviving bacteria.

### Determination of the Minimum Antibiotic Concentration of Killing (MCK) and the Biofilm Tolerance Factors

Antibiotic concentration-dependent killing of biofilm-grown cells was determined based on the reduction of CFUs following the addition of different antibiotic concentrations as compared to an untreated control well. The number of surviving bacteria for each clinical isolate for overall six different concentrations of the three different antibiotics was recorded. From those data the minimum antibiotic concentrations of killing (MCK) that resulted in a CFU reduction of biofilm-grown cells of at least 1-log, 2-log, 3-log, 4-log, 5-log, 6-log or eradication (below the detection limit) could be deduced.

To quantify biofilm-induced tolerance of the individual isolates, the biofilm tolerance factor (BTF) was introduced. The BTF results from the ratio of the concentration that is required to achieve a certain log-CFU reduction in biofilm-grown cells, and the respective MIC of the strain:


$$\text{BTF}=\frac{\text{MCK (CFU log}-\text{reduction})}{\text{MIC}}$$

BTF: Biofilm tolerance factor; MCK(CFU log-reduction): minimum antibiotic concentration of killing at which a certain log-reduction [1 log (BTF-1), 2 log (BTF-2), 3 log (BTF-3), 4 log (BTF-4), 5 log (BTF-5), or 6 log (BTF-6) units as well as eradication (BTF-E)] in the CFUs of biofilm-grown cells was observed; MIC: minimal inhibitory concentration

### Correlation Analyses and Hierarchical Clustering Using Biofilm Tolerance Factors

An integrated analysis of biofilm tolerance factors (BTFs) for the three antibiotics tested was used to uncover cross-tolerance and characteristic patterns in the tolerance behavior of biofilm-grown cells. Only discrete BTFs were used, where the highest antibiotic concentration tested (1,024 µg/ml) resulted in a measurable reduction of CFUs. BTFs were log2-transformed prior to further analyses.

Correlation analyses were performed in order to access cross-tolerance. Correlation coefficients for log2-transformed BTFs for the different antibiotics were calculated pairwise using the Correlation analysis tool in Microsoft Excel. Simple linear regression was calculated and plotted in GraphPad Prism (v 8.3.0). A hierarchical clustering approach was performed using the web tool ClustVis (Metsalu and Vilo, 2015) to group clinical isolates (n = 352) into six clusters of strains with characteristic biofilm tolerance patterns. Log2-transformed BTFs for all three antibiotics and all possible log-reductions were used as input data resulting in a heatmap of 352 rows (clinical isolates) and 21 columns (BTFs). No scaling was applied to rows. Rows were clustered using Euclidian distance and Ward linkage.

## Results

### Antimicrobial Susceptibility Testing of Planktonic and Biofilm-Grown Clinical *P. aeruginosa* Isolates

Antimicrobial susceptibility testing of planktonic as well as biofilm-grown bacterial cells was performed on a collection of overall 352 clinical *P. aeruginosa* strains isolated from various infection sites (**Electronic Supplementary Table ES1**). While the minimal inhibitory concentration (MIC) values of the planktonically grown cells have been determined in a previous study (Khaledi et al., 2020) and are publicly available (Hornischer et al., 2019), we recorded antimicrobial activities on biofilm-grown bacteria in this study. We restricted our analysis to the anti-biofilm activity of tobramycin (an aminoglycoside), ciprofloxacin (a fluoroquinolone), and colistin (a polymyxin), all of which are frequently used to treat chronic *P. aeruginosa* infections e.g. of the respiratory tract of cystic fibrosis patients (Banerjee and Stableforth, 2000). Based on their MIC values and according to the CLSI guidelines (Weinstein et al., 2021), the great majority of isolates included in this study was classified as susceptible to tobramycin (86 % of the 352 clinical isolates) and colistin (82 %), while 44 % of the strains were ciprofloxacin-susceptible (**Supplementary Figure 1**; **Supplementary Table 1**).

**Figure 1:**
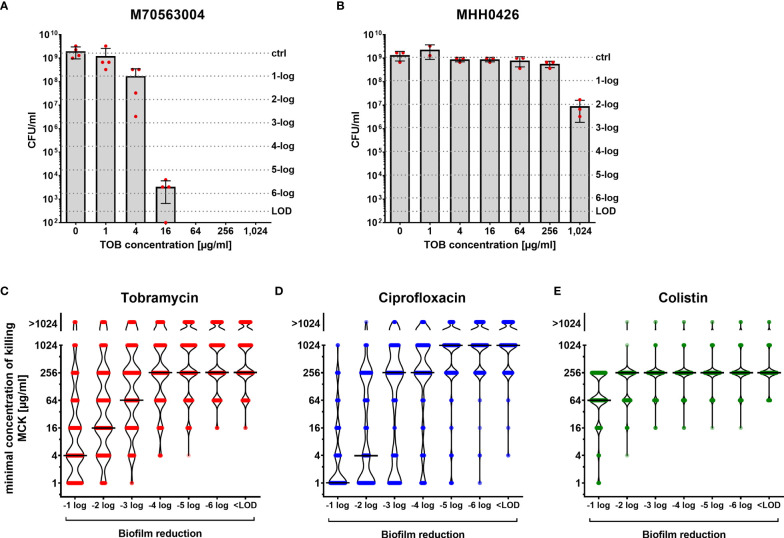
Concentration-dependent killing of biofilm-grown clinical *P. aeruginosa* isolate cells. Isolate-specific killing profiles are exemplarily shown for two tobramycin susceptible clinical isolates, which exhibit the same minimal inhibitory concentration (MIC) value (MIC = 1 µg/ml). M70563004 biofilm-grown cells are responsive to tobramycin treatment **(A)**, whereas MHH0426 biofilm-grown cells are far less responsive **(B)**. Mean and standard deviations of the CFU/ml following the antibiotic treatment of biofilm-grown cells at the indicated tobramycin concentrations for 24 h are depicted. Results of the individual independent experiments are indicated by red dots (n = 4 for M70563004; n = 3 for MHH0426). The antibiotic concentration (minimal concentration of killing, MCK) that was required to lead to a 1-log, 2-log, 3-log, 4-log, 5-log, and 6-log unit reduction (dashed lines) in colony forming units (CFU) as compared to the untreated growth control can be deduced. The violin plots **(C–E)** depict the minimal concentrations of killing (MCK) to achieve a 1-log, 2-log, 3-log, 4-log, 5-log, 6-log unit reduction in colony forming units (CFU), and eradication below the limit of detection (< LOD), respectively for all 352 clinical isolates. Black lines indicate the median MCKs of tobramycin **(C)**, ciprofloxacin **(D)** and colistin **(E)**. Each dot represents one clinical isolate. TOB, tobramycin; CIP, ciprofloxacin; COL, colistin; LOD, limit of detection.

We found that the different clinical isolates exhibit a very wide distribution of susceptibility to the three antibiotics when grown under biofilm conditions, even when they exhibited similar antimicrobial susceptibility profiles (MIC values) under planktonic conditions. Some clinical *P. aeruginosa* isolates proved to be remarkably responsive towards antimicrobial killing and already low antibiotic concentrations resulted in a significant reduction of biofilm-grown cells (**Figure 1A**). However, other isolates were largely non-responsive and the numbers of surviving biofilm-grown cells decreased only at very high antibiotic concentrations (**Figure 1B**).

**Figures 1C–E** depict the killing efficiency of the three antibiotics (tobramycin, ciprofloxacin, and colistin) on all 352 biofilm-grown clinical isolates. The minimum antibiotic concentration of killing (MCK) at which the colony-forming units (CFUs) of biofilm-grown bacteria was reduced by 1-log, 2-log, 3-log, 4-log, 5-log and 6-log units compared to growth of the untreated control in the individual isolates, as well as eradicated below the detection limit, is depicted (**Figures 1C–E**; **Electronic Supplementary Table ES1**). In general, higher reductions in the CFUs of biofilm-grown cells required higher concentrations of the antibiotic. For tobramycin, a gradual increase in the median MCKs was observed, indicating that the killing efficiency increases proportionally with the antibiotic concentration (**Figure 1C**). In contrast, we observed a disproportional increase in the median MCKs for ciprofloxacin (**Figure 1D**). While ciprofloxacin seems to be quite potent to achieve low (2-log) reductions in biofilm-grown clinical isolates at low antibiotic concentrations, much higher concentrations had to be applied to achieve reductions of ≥ 3 logs across the isolates. The profile was different again with colistin. We observed that a concentration of 256 µg/ml was required to achieve a 2-log reduction in CFUs in the vast majority of biofilm-grown strains (**Figure 1E**). However, once this colistin concentration threshold (256 µg/ml) was reached, the eradication of biofilm-grown cells across the clinical isolates became largely concentration-independent (all-or-none phenomenon).

Of note, several clinical isolates showed a remarkable biofilm recalcitrance, particularly towards tobramycin and ciprofloxacin. Even the highest concentration applied in this screening (1,024 µg/ml of the antibiotic) was not sufficient to reduce the CFUs of biofilm-grown cells (**Figures 1C–E**).

### Strains With Higher Ciprofloxacin and Tobramycin MICs Require Higher Antimicrobial Concentrations to Reduce the CFUs of Biofilm-Grown Cells

**Figure 2:**
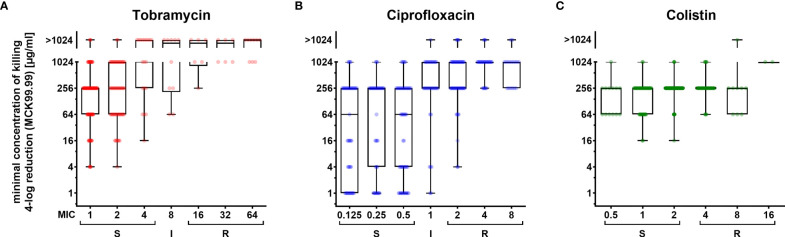
Concentration-dependent killing of biofilm-grown clinical *P. aeruginosa* isolate cells exhibiting different resistance levels. Minimum concentrations of killing (MCK) for tobramycin **(A)**, ciprofloxacin **(B)** and colistin **(C)** that are required to reduce biofilm-grown cells by 99.99 % (4-log reduction of CFU; MCK99.99) are depicted in box-plots for clinical isolates, which were categorized into sub-groups exhibiting the same MIC values (x-axis). Boxplot elements are: center line – median; box limits – upper and lower quartiles; whiskers – minimum and maximum. Each dot represents one clinical isolate. S, susceptible; I, intermediate; R, resistant. Strains were categorized according to breakpoints defined by CLSI guidelines.

Our biofilm susceptibility screening has shown that the higher the antibiotic concentrations, the more pronounced are the CFU reductions across the biofilm-grown clinical isolates. However, we also observed that the minimum concentrations of killing (MCK) required for a certain reduction in CFUs were very different across the individual clinical strains. To evaluate whether killing efficiency is dependent on the underlying MIC, we sorted the 352 clinical strains according to their MIC values and depicted the MCKs for those clinical strains that exhibited a MIC around the CLSI breakpoints (**Figure 2**). For the vast majority of clinical isolates, we recorded MCKs far above their MICs, confirming the generally higher recalcitrance of biofilm-grown cells as compared to their planktonic counterparts (**Figure 2**; **Supplementary Figure 2**). Interestingly, in general – despite the very broad distribution of MCKs even in clinical isolates exhibiting the same MIC – the median MCK99.99 (antibiotic concentrations required for a 4-log CFU reduction, **Figures 2A, B**) and the median MCK99 (required for a 2-log reduction, **Supplementary Figures 2A, B**) gradually increased in clinical isolates that exhibited increased MICs for tobramycin and ciprofloxacin. Thus, the proportion of strains that did not respond to the highest concentration used in this screening (1,024 µg/ml tobramycin or ciprofloxacin) was highest among resistant strains (**Supplementary Figures 2D, E**), while strains responsive to low concentrations of antibiotics under biofilm growth conditions, were almost exclusively found among strains exhibiting low MIC values. As opposed to ciprofloxacin and tobramycin, colistin-mediated killing of biofilm-grown cells was found to act independently of the MIC of the respective individual strains. The MCK99, MCK99.99 and the 6-log kill rate were stable (256 µg/ml) for most of the strains despite differing MICs (**Figure 2C**; **Supplementary Figures 2C, F**).

### Biofilm-Induced Tolerance Versus Antimicrobial Resistance in the Individual Clinical Isolates

**Figure 3:**
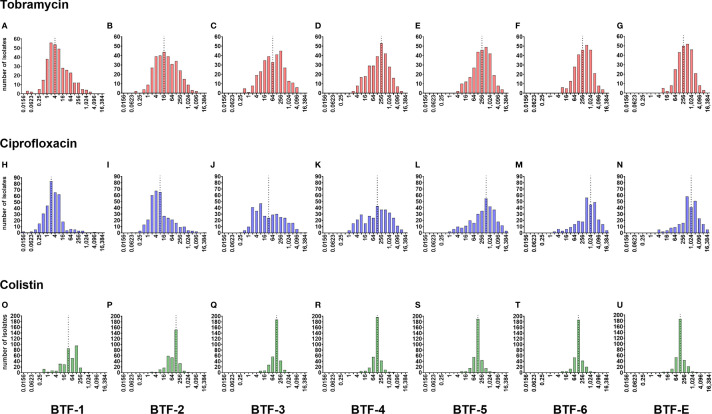
Histograms of the biofilm tolerance factors (BTFs) of the individual clinical isolates. Distributions of biofilm tolerance factors (BTF) by which the minimal inhibitory concentration (MIC) has to be multiplied to achieve a reduction in the CFUs of the individual biofilm-grown clinical isolates by 1-log (BTF-1), 2-log (BTF-2), 3-log (BTF-3), 4-log (BTF-4), 5-log (BTF-5), 6-log (BTF-6) reduction and eradication below the detection limit (BTF-E), respectively, are shown for tobramycin **(A–G)**, ciprofloxacin **(H–N)**, and colistin **(O–U)**. Tested clinical isolates: n = 352. BTF = MCK/MIC. Graphs only include strains with discrete BTF, where the highest antibiotic concentration tested (1,024 µg/ml) resulted in the indicated reduction of biofilm CFU. Median biofilm tolerance factors are indicated by a dashed line.

It is commonly claimed that biofilm-grown bacteria exhibit an up to 1,000-fold increased tolerance towards antibiotics as compared to their planktonically grown counterparts. In order to provide a better data basis, we determined the biofilm tolerance factor (BTF) of the clinical isolates against the three tested antibiotics. The BTF indicates which multiple of antibiotic concentration under biofilm-growth conditions as compared to the MIC (determined under planktonic growth conditions) must be used to achieve a CFU reduction of at least 1 log (BTF-1), 2 log (BTF-2), 3 log (BTF-3), 4 log (BTF-4), 5 log (BTF-5), 6 log (BTF-6) units or to observe eradication (BTF-E) below the detection limit (**Electronic Supplementary Table ES1**). Histograms of the BTFs of the individual clinical isolates are shown in **Figure 3**. Of note, BTFs could only be determined for strains for which a concentration of 1,024 µg/ml was sufficient to reach the respective log-reduction in the CFUs of biofilm-grown cells (**Supplementary Table 1**).

We found that for 1-log CFU reductions the median biofilm tolerance factor (BTF-1) across the clinical isolates was 4 for tobramycin, 2 for ciprofloxacin and 32 for colistin. This factor shifted to a median BTF-E (biofilm tolerance factor for eradication) of 256 for tobramycin, and 1,024 for ciprofloxacin, while it reached the maximum of 128 for colistin already at BTF-2 (**Figure 3**). Thus, depending on the antibiotic, this factor varies substantially, and is additionally greatly influenced by the *P. aeruginosa* strain background. We identified a small number of *P. aeruginosa* isolates, in which an antibiotic concentration around the MIC (of ciprofloxacin and less frequently tobramycin) was sufficient to achieve a 2-log (99 %) CFU reduction of biofilm-grown bacteria (**Figures 3A, H**). However, there were also isolates for which antibiotic concentrations of > 4,000 times the MIC for tobramycin or > 16,000 times the MIC for ciprofloxacin, were required to achieve a 2-log reduction in CFUs of biofilm-grown bacteria.

Our data also illustrate that the distribution of the BTFs among the clinical isolates was different in the three antibiotics tested. The fraction of clinical isolates that were effectively reduced by 2-log already at a low BTF was highest for ciprofloxacin. However, the proportion of isolates that required a high BTF to achieve a substantial reduction in the CFU of biofilm-grown bacteria was also highest for ciprofloxacin (**Figures 3H–N**). The opposite was found for colistin. Most clinical isolates required already a high BTF to achieve even a 2-log CFU reduction (**Figure 3P**). However, on the other end of the scale more clinical isolates were eradicated with overall only slightly higher colistin BTFs (**Figure 3U**). It seems that once a certain colistin killing concentration is reached, this concentration seems to efficiently eradicate biofilm-grown cells in a concentration-independent manner and independent on the resistance background within the clinical isolate.

### Cross-Tolerance of the Clinical Isolates Against Ciprofloxacin and Tobramycin

**Figure 4:**
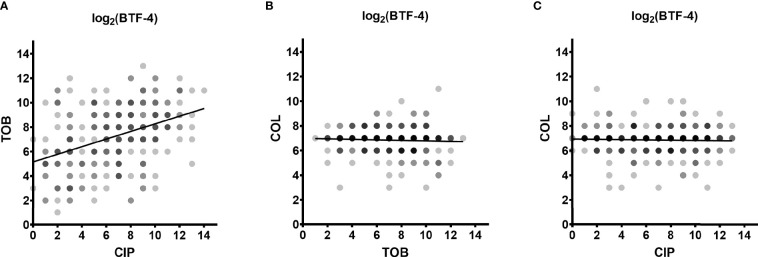
Cross-tolerance of biofilm-grown cells. The dependence between the log2-transformed BTF-4 of the three antibiotics on all clinical isolates is depicted. The correlation coefficient between the BTF-4 of tobramycin (TOB) and ciprofloxacin (CIP) was 0.4148 **(A)**. No correlation was observed between the BTF-4 of colistin (COL) and TOB **(B)** or CIP **(C)**. BTF-4 - minimum antibiotic concentration killing 99.99 % of the biofilm-grown cells (MCK99.99; 4-log reduction) divided by the minimal inhibitory concentration (MIC) of the individual isolate. Dots represent the log2-transformed BTF-4 of the individual clinical isolates. Darker shades indicate overlapping datapoints.

In order to determine whether there is cross-tolerance in individual clinical biofilm-grown isolates towards the activity of the three antibiotics, we performed correlation analyses. While we found there was no correlation between colistin BTFs and those of ciprofloxacin or tobramycin, there was a positive correlation (correlation coefficient of 0.4148) between the killing efficiency of ciprofloxacin and tobramycin (reduction of 99.99 % of the biofilm-grown cells (BTF-4), **Figure 4**). We observed a similar trend for smaller (BTF-2) and larger log-CFU reductions (BTF-6) (**Supplementary Figure 3**).

### The Higher the Ciprofloxacin and Tobramycin MIC Values, the Lower the BTFs

**Figure 5:**
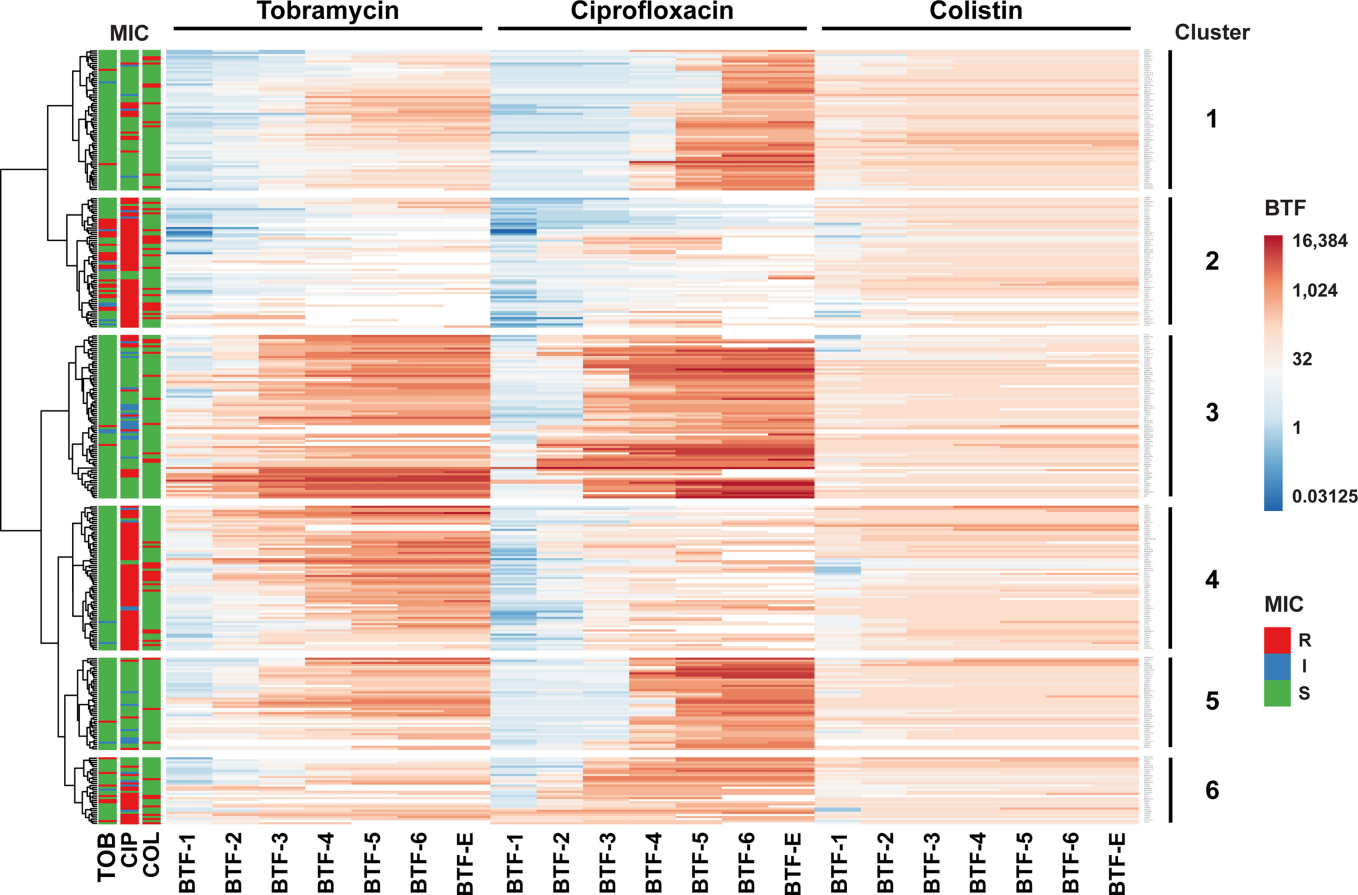
Hierarchical clustering of biofilm tolerance factors. The 352 clinical isolates were clustered based on their biofilm tolerance factors for all three antibiotics. The result of the hierarchical clustering calculation is displayed in a heat map and uncovers six groups of strains (indicated on the right) that exhibit similar tolerance patterns. Hierarchical clustering was based on log2-transformed BTFs, using Euclidian distance and Ward linkage in ClustVis (Metsalu and Vilo, 2015). Only discrete BTFs are shown, where the highest antibiotic concentration tested (1,024 µg/ml) resulted in a measurable reduction of CFUs. The MIC-based susceptibility profiles of clinical isolates (according to CLSI breakpoints) are displayed on the left. R, resistant (marked in red); I, intermediate (blue); S, susceptible (green).

In order to characterize the conditional tolerance phenotype of the individual clinical isolates in more detail, we integrated all data on the BTFs of the three antibiotics and performed a hierarchical cluster analysis across all clinical isolates. We identified six clusters of clinical strains with characteristic patterns in their biofilm tolerance factors (**Figure 5**; **Electronic Supplementary Table ES1**). Clinical isolates in cluster 2 and in cluster 4 generally exhibited lower ciprofloxacin BTFs, whereas isolates in cluster 2 exhibited additionally lower tobramycin BTFs. Interestingly, a majority of strains assigned to cluster 4 exhibited elevated ciprofloxacin MICs, whereas the isolates in cluster 2 exhibited elevated ciprofloxacin as well as tobramycin MICs. This result shows that strains that have inherited genetically encoded resistance mechanisms to tobramycin and/or ciprofloxacin show an increase in overall tolerance under biofilm-growth conditions that is less pronounced compared to the increase in tolerance observed in clinical strains with low MIC values.

## Discussion

In order to get a full picture of biofilm-growth mediated tolerance phenotypes in the opportunistic pathogen *P. aeruginosa*, we investigated the efficiency of three different classes of antibiotics to kill biofilm-grown cells in a large collection of diverse clinical isolates (n = 352). Those have been isolated from various infection sites and from acute and chronic infections. As opposed to previous studies, which had introduced the minimum duration of killing (MDK) as a quantitative indicator for tolerance of planktonic cultures in time-resolved killing experiments (Brauner et al., 2016), we determined the minimum antibiotic concentration of killing (MCK) as a quantitative measure of biofilm-induced tolerance in concentration-dependent killing experiments. Typically, MDK99 values (time to kill 99 % of the bacterial cells) are used to describe tolerance on a population level. Here, we recorded the MCK99 value (antibiotic concentration required to kill 99 % of the bacterial cells) and additionally the MCK90, MCK99.9, MCK99.99, MCK99.999, MCK99.9999 as well as the MCK that is required to eradicate biofilm-grown cells (below the detection limit).

Our data showed first of all, that the MCKs were impacted to a very large extent by the individual strain background. We found very susceptible clinical isolates that were killed by a comparatively low antibiotic concentration under biofilm-growth conditions, while other strains were only effectively killed after exposure to very high antibiotic concentrations. For some strains, up to 8,000 times the MIC for tobramycin and up to 16,000 times the MIC for ciprofloxacin were required in order to kill 99.99 % of the biofilm-grown cells. Interestingly, this inter-strain variation of the MCKs was very much dependent on the antibiotic. The highest variation of the MCKs among the individual clinical isolates was observed for ciprofloxacin. While a ciprofloxacin concentration of 4 µg/ml or less killed 99 % of the biofilm cells in more than half of the tested isolates (54 %), a concentration of 256 µg/ml or more was required to achieve the same biofilm killing in 31 % of the isolates.

Variation in tobramycin MCKs was lower, while the clinical isolates responded most consistently to colistin. The great majority of the clinical isolates (90 %) were eradicated at a colistin concentration of 256 µg/ml. While this concentration was required to kill at least 90 % of the biofilm-grown bacterial population, the same colistin concentration was also sufficient to eradicate the biofilm-grown cells to levels below the detection limit.

Our observation of a broad distribution of the MCK values across the clinical isolates is remarkable and suggests that while the biofilm growth mode in itself is sufficient to explain tolerance in clinical isolates, the respective tolerance levels seem to be largely modulated by the individual strain background. Interestingly, biofilm-induced tolerance against ciprofloxacin and tobramycin, but not colistin, were correlated in the individual clinical isolates. This result underlines that the conditional tolerance phenotype seems to be non-specific and is determined both, by the biofilm growth pattern and by additional isolate-specific, tolerance-promoting mechanisms that help bacteria survive transient exposure to high antibiotic concentrations. Despite extensive research on the identification of bacterial factors that determine tolerance-promoting phenotypes, we are only at the beginning to understand the molecular mechanisms that drive bacterial tolerance. It seems that changes in the activity of the tricarboxylic acid (TCA) cycle, cellular respiration, the proton motive force as well as shifts in the intracellular pH drive bacterial tolerance phenotypes (Allison et al., 2011; Meylan et al., 2017; Crabbé et al., 2019; Donnert et al., 2020; Zheng et al., 2020; Arce-Rodríguez et al., 2022). Furthermore, it is conceivable that additional factors contribute to biofilm tolerance phenotypes, such as restricted penetration of the antibiotic through the biofilm matrix, a biofilm-specific expression of resistance genes, or the presence of a sub-population of dormant persister cells (Stewart, 1996; Hentzer et al., 2001; Lewis, 2001; Pamp et al., 2008; Zhang and Mah, 2008; Chiang et al., 2013; Hall and Mah, 2017).

Despite the large variability in the MCKs, our extensive data on many clinical isolates allowed the detection of a positive correlation between the MIC values of the individual isolates and the concentration of antibiotics (MCK) that was required to kill biofilm-grown cells. These results suggest that genetically inherited resistance mechanisms affect the biofilm-induced tolerance phenotype. In general, strains found to be resistant as categorized by elevated MIC levels also required high concentrations of antibiotics to achieve biofilm reduction of 4-logs or more. A synergistic interaction between tolerance and resistance has been described previously (Levin-Reisman et al., 2019) and it has been speculated that genetically inherited resistance factors might be more effective in slow growing cells, and thus might act synergistically with tolerance to protect biofilm-grown cells.

Strikingly, at the same time we observed that the strains exhibiting high MIC values seemed to express lower biofilm-specific tolerance factors (BTFs) as defined by the multiple of the MIC concentration required to kill the biofilm-grown population. Thus, it seems that once a certain threshold concentration of an antibiotic is reached, biofilm-grown bacteria are efficiently killed, independent on their individual genetically determined resistance profile. A recent study demonstrated that antibiotic exposed strains developed either high-level multidrug tolerance or antibiotic resistance, but not both (Santi et al., 2021). It could be argued that once high-level tolerance is reached, additional genetically determined resistance mechanisms do not add further to survival.

It has already been shown several times that antibiotic susceptibility profiles of biofilm-grown cells cannot be predicted on the basis of MIC profiles. Thus MIC determinations cannot serve as an approximation on which is the best therapy to apply to treat patients suffering from chronic biofilm-associated diseases (Moskowitz et al., 2004; Keays et al., 2009; Waters and Ratjen, 2015; Brady et al., 2017; Müsken et al., 2017; Coenye et al., 2018). However, our study also revealed that leveraging drug-specific properties might have great potential to optimize future treatments of biofilm-associated infections. If a certain threshold concentration of colistin can be reached in e.g. the respiratory tract of CF patients, then not only the great majority of the clinical isolates, independent on their individual strain background, might be efficiently killed, but this killing also becomes concentration independent. It appears that colistin overcomes the conditional tolerance mechanisms of the individual clinical strains, and thus, as suggested before (Pamp et al., 2008; Elborn et al., 2009; Kolpen et al., 2016), it might be a valuable therapeutic option for chronic biofilm-associated infections characterized by the presence of particularly tolerant strains.

## Data Availability Statement

The original contributions presented in the study are included in the article/**Supplementary Material**. Further inquiries can be directed to the corresponding author.

## Author Contributions

JT, conception and design, acquisition and curation of the data, analysis and interpretation of the data, drafting and revising the manuscript. SH, conception and design, analysis and interpretation of the data, drafting and revising the manuscript, funding acquisition. All authors have read and agreed to the published version of the manuscript.

## Funding

SH was funded by the European Union (EU, ERC Consolidator Grant COMBAT 724290) and received funding as part of the excellence cluster RESIST (Resolving Infection Susceptibility; EXC 2155). Furthermore, SH received funding from the German Research Foundation (DFG SPP 1879) and the Novo Nordisk Foundation (NNF 18OC0033946).

## Conflict of Interest

The authors declare that the research was conducted in the absence of any commercial or financial relationships that could be construed as a potential conflict of interest.

## Publisher’s Note

All claims expressed in this article are solely those of the authors and do not necessarily represent those of their affiliated organizations, or those of the publisher, the editors and the reviewers. Any product that may be evaluated in this article, or claim that may be made by its manufacturer, is not guaranteed or endorsed by the publisher.
