# Supplementary material for: Pseudomonas aeruginosa Is More Tolerant Under Biofilm Than Under Planktonic Growth Conditions: A Multi-Isolate Survey
Source: Front Cell Infect Microbiol. 2022 Feb 28;12:851784. doi: 10.3389/fcimb.2022.851784 (PMC8920030; doi:10.3389/fcimb.2022.851784)
Supplement: Supplementary file 1 [file DataSheet_1.pdf]

Supplementary Material

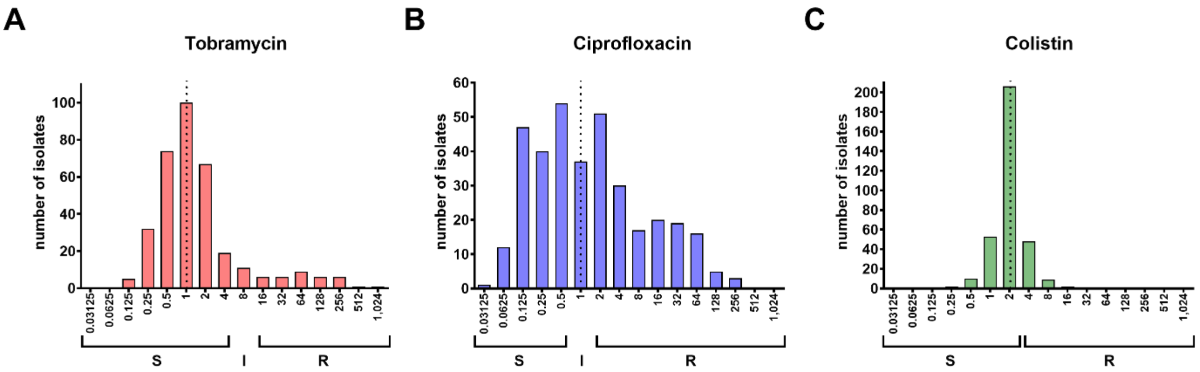

Supplementary Figure 1: Distribution of minimal inhibitory concentrations (MIC) for clinical *P. aeruginosa* isolates. Histograms of the MIC values of the 352 clinical isolates against tobramycin (**A**), ciprofloxacin (**B**), and colistin (**C**). S – susceptible; I – intermediate; R – resistant. Strains were categorized according to breakpoints defined by CLSI guidelines. Median MIC values are indicated by a dashed line.

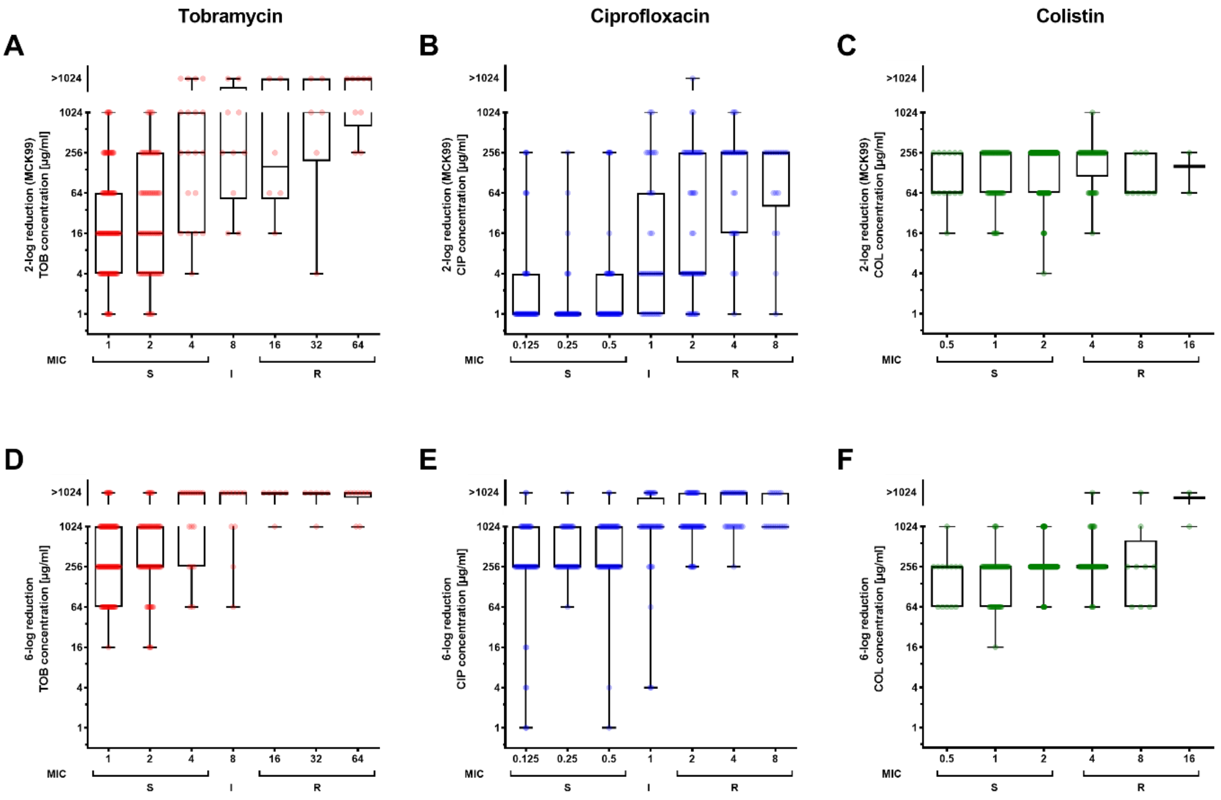

Supplementary Figure 2: Concentration-dependent killing of biofilm-grown clinical *P. aeruginosa* isolate cells exhibiting different resistance levels. Minimum concentrations of killing (MCK) for tobramycin (**A, D**), ciprofloxacin (**B, E**) and colistin (**C, F**) that are required to reduce biofilm-grown cells by 99 % (2-log reduction of CFU; MCK99; **A-C**) and those that are required to reduce biofilm-grown cells by 6-log (**D-F**) are depicted in box-plots. The clinical isolates were categorized into sub-groups exhibiting the same MIC values (x-axis). Boxplot elements are: center line – median; box limits – upper and lower quartiles; whiskers – minimum and maximum. Each dot represents one clinical isolate. S – susceptible; I – intermediate; R – resistant. Strains were categorized according to breakpoints defined by CLSI guidelines.

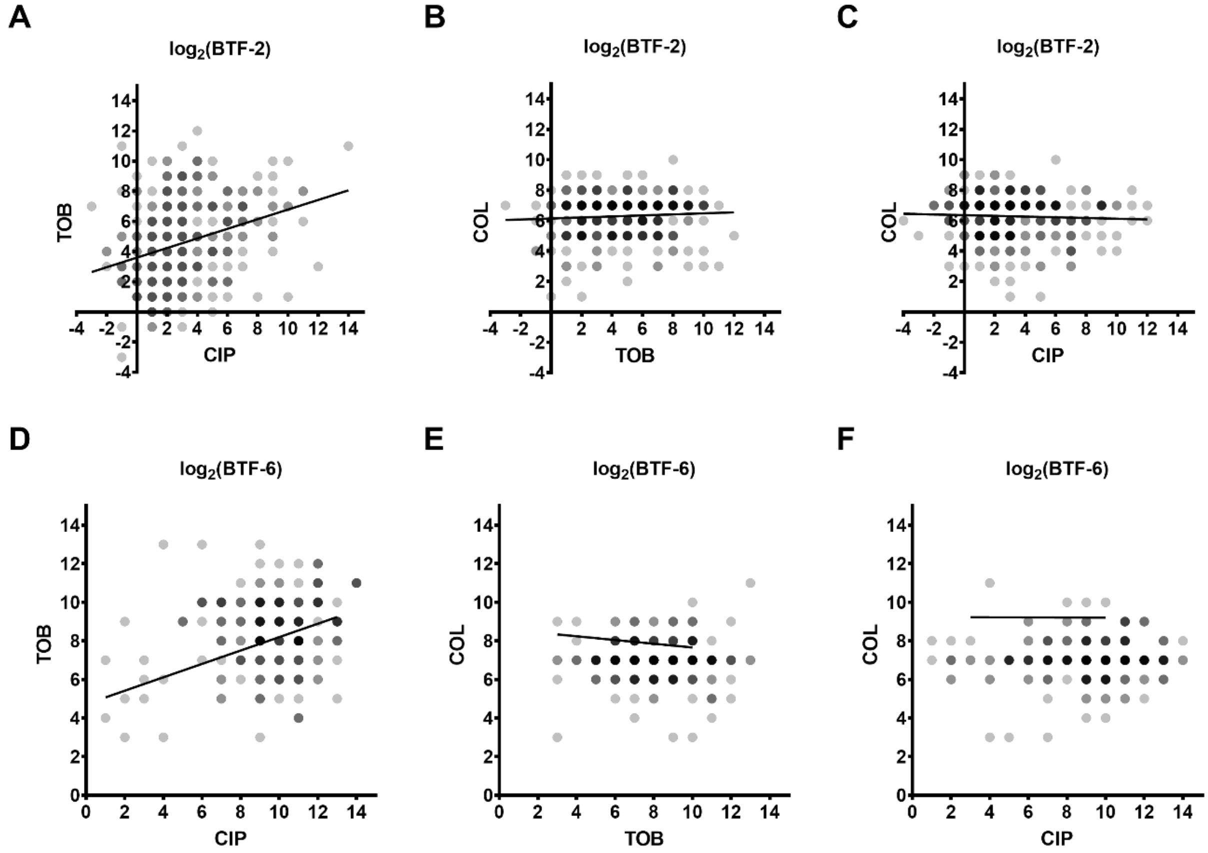

Supplementary Figure 3: Cross-tolerance of biofilm-grown cells. The dependence between the log2-transformed BTF-2 (**A-C**) and BTF-6 (**D-F**) of the three antibiotics on all clinical isolates is depicted. The correlation coefficient between the BTF-2 of tobramycin (TOB) and ciprofloxacin (CIP) was 0.3159 (**A**), and the correlation coefficient between the BTF-6 of tobramycin (TOB) and ciprofloxacin (CIP) was 0.1987 (**D**). No correlation was observed between the BTF-2 or the BTF-6 of colistin (COL) and TOB (**B, E**) or CIP (**C, D**). Dots represent the log2-transformed BTF-2 and BTF-6 respectively of the individual clinical isolates. Darker shades indicate overlapping datapoints.

Supplementary Table 1: Antimicrobial susceptibility testing for 352 clinical *P. aeruginosa* isolates in planktonic and biofilm growth conditions. MIC – minimal inhibitory concentration; MCK – minimum antibiotic concentration of killing to achieve a certain reduction of biofilm colony-forming units (CFU); BTF – biofilm tolerance factor by which the MIC has to be multiplied to achieve a certain reduction of biofilm CFUs (BTF = MCK / MIC); min – lowest concentration/factor detected for at least one strain in the collection; max – highest concentration/factor detected; n(valid) – number of strains for which a conclusive test result was observed; n(n.d.) – number of strains for which the respective MCK and BTF respectively could not be exactly determined because the highest concentration tested (1,024 µg/ml) was not sufficient to result in the indicated reduction of biofilm CFU. S – susceptible; I – intermediate; R – resistant (according to CLSI guidelines). Concentrations are depicted in [µg/ml]. Concentrations ranging from 1 to 1,024 were used to test biofilm-induced tolerance.

		Minimal inhibitory concentration (MIC)	Minimum antibiotic concentration of killing (MCK)							Biofilm tolerance factor (BTF)						
			MCK90 -1 log	MCK99 -2 log	MCK99.9 -3 log	MCK99.99 -4 log	MCK99.999 -5 log	MCK99.9999 -6 log	MCK-E eradication	BTF-1 -1 log	BTF-2 -2 log	BTF-3 -3 log	BTF-4 -4 log	BTF-5 -5 log	BTF-6 -6 log	BTF-E eradication
**Tobramycin**	n(valid) =	352	341	339	334	333	337	338	339	341	339	334	333	337	338	339
	n(n.d.) =	-	10 (2.9 %)	20 (5.9 %)	32 (9.6 %)	46 (13.8 %)	60 (17.8 %)	72 (21.3 %)	79 (23.3 %)	10 (2.9 %)	20 (5.9 %)	32 (9.6 %)	46 (13.8 %)	60 (17.8 %)	72 (21.3 %)	79 (23.3 %)
	min	0.125	1	1	1	4	4	16	16	0.03125	0.125	0.5	2	4	8	8
	max	1,024	>1,024	>1,024	>1,024	>1,024	>1,024	>1,024	>1,024	2,048	4,096	4,096	8,192	8,192	8,192	8,192
	median	1	4	16	128	256	256	256	256	4	16	64	256	256	256	256
	S	303 (86 %)														
	I	14 (4 %)														
	R	35 (10 %)														
**Ciprofloxacin**	n(valid) =	352	352	350	347	347	349	350	351	352	350	347	347	349	350	351
	n(n.d.) =	-	0 (-)	2 (0.6 %)	10 (2.9 %)	22 (6.3 %)	44 (12.6 %)	84 (24 %)	101 (29 %)	0 (-)	2 (0.6 %)	10 (2.9 %)	22 (6.3 %)	44 (12.6 %)	84 (24 %)	101 (29 %)
	min	0.03125	1	1	1	1	1	1	4	0.015625	0.0625	0.25	1	1	2	4
	max	256	1,024	>1,024	>1,024	>1,024	>1,024	>1,024	>1,024	4,096	16,384	16,384	16,384	16,384	16,384	16,384
	median	1	1	4	256	256	1,024	1,024	1,024	2	8	32	128	512	1,024	1,024
	S	154 (44 %)														
	I	37 (10 %)														
	R	161 (46 %)														
**Colistin**	n(valid) =	352	337	337	337	337	333	330	330	337	337	337	337	333	330	330
	n(n.d.) =	-	0 (-)	1 (0.3 %)	2 (0.6 %)	2 (0.6 %)	2 (0.6 %)	4 (1.2 %)	4 (1.2 %)	0 (-)	1 (0.3 %)	2 (0.6 %)	2 (0.6 %)	2 (0.6 %)	4 (1.2 %)	4 (1.2 %)
	min	0.25	1	4	16	16	16	16	64	0.5	2	8	8	8	8	8
	max	32	256	>1,024	>1,024	>1,024	>1,024	>1,024	>1,024	1,024	1,024	1,024	2,048	2,048	2,048	2,048
	median	2	64	256	256	256	256	256	256	32	128	128	128	128	128	128
	S	287 (82 %)														
	R	65 (18 %)														
